# ADHD POLYGENIC RISK AND COGNITIVE PERFORMANCE IN LATER LIFE

**DOI:** 10.1093/geroni/igac059.2249

**Published:** 2022-12-20

**Authors:** Marrium Mansoor, Benjamin Katz

**Affiliations:** Virginia Tech Gradueate School, Blacksburg, Virginia, United States; Virginia Tech, Blacksburg, Virginia, United States

## Abstract

Attention-Deficit/Hyperactivity Disorder (ADHD) affects approximately 4.4% of adults in the US and impacts multiple domains of daily life including education, workplace performance and interpersonal relationships. Although an increasing number of individuals with ADHD are now entering later life, there is very little research on how ADHD risk may impact cognitive function during aging. As such, there is value in understanding the association between polygenic risk for ADHD and cognition during different stages of later life. This study utilized data from the Health and Retirement Study which surveys 37,000 Americans biennially and aimed to determine if there was an influence of ADHD risk on cognitive performance when individuals were young-old (ages 65-74) or middle-old (ages 75-84). Only participants who responded in 2006, 2016, and to the Venous Blood Study were selected. The resulting sample size of 403 African-ancestry individuals (AA) and 2286 European-ancestry individuals (EA) was compared on executive function-focused measures as well as delayed recall measures. Results showed that there was no significant effect of ADHD risk on memory-related measures at both time-points for AA and EA individuals. However, there was a statistically significant association between ADHD risk and performance on the executive function measure for EA older adults who were middle-old (p = 0.028), but not when they were young-old; no such association was observed for AA adults. This finding suggests that ADHD risk may influence cognition among older adults and has significant implications for treatment and care of individuals with ADHD throughout the life course.

